# Assessment of Perceptions of Human Papillomavirus (HPV) Vaccine Among Japanese Healthcare Professional University Students Using Text Mining Analysis

**DOI:** 10.7759/cureus.72598

**Published:** 2024-10-29

**Authors:** Akihiro Yokoyama, Hiromi Suzuki, Hiroaki Kataoka, Nobuhiro Nasu, Yoshiro Mori, Yuji Watanabe, Rumi Nohara, Nobuyuki Miyatake

**Affiliations:** 1 Department of Hygiene, Faculty of Medicine, Kagawa University, Miki, JPN; 2 Department of Physical Therapy, Faculty of Health Sciences, Okayama Healthcare Professional University, Okayama, JPN; 3 Department of Public Health, Graduate School of Biomedical Sciences, Tokushima University, Tokushima, JPN; 4 Department of Occupational Therapy, Faculty of Health Sciences, Okayama Healthcare Professional University, Okayama, JPN; 5 Department of Nursing, Faculty of Medicine, Kagawa University, Miki, JPN

**Keywords:** cervical cancer, correspondence analysis, hpv vaccine, text mining, university student

## Abstract

Objective: Human papillomavirus (HPV) vaccination is crucial, particularly for preventing cervical cancer. This study aimed to assess the perceptions of HPV vaccination among healthcare university students in Okayama City, Japan, with the goal of promoting HPV vaccination in the future.

Methods: The study enrolled 168 students (94 male students and 74 female students, median age: 20 (minimum: 18, maximum: 27)) from a healthcare university in Okayama, Japan. Data collected included sex, age, year, HPV vaccination status, knowledge about HPV vaccination, and cervical cancer screening status (for female students). Participants completed self-administered questionnaires on their perceptions of HPV vaccination. The responses were analyzed using text mining.

Results: The HPV vaccination rate among female participants was 16.2% (12 out of 74). Of the respondents, 43.6% of male respondents and 52.7% of female respondents knew that HPV causes cervical cancer. Text mining revealed that among the female respondents, the most frequently used words were “think”, followed by “vaccine”, “side effects”, “vaccination”, and “frightening”. Distinctive words among female respondents, especially those who haven't been vaccinated, include knowing the term "HPV," understanding that HPV causes cervical cancer, and for those who haven't had screenings, terms like "side effects" and " frightening" were common.

Conclusion: Among female students at the healthcare university, the HPV vaccination rate was thought to be comparatively low. Among those who had not received the HPV vaccine, it was particularly noted that they knew the term "HPV," were aware that HPV caused cervical cancer, and for those who had not undergone screenings, providing accurate information about "side effects" and " frightening" seemed necessary.

## Introduction

Cervical cancer has become a public health challenge in the world, and it is the fourth most common cancer in terms of incidence and mortality among women worldwide [1]. In 2022, there were an estimated 660,000 new cases and 350,000 deaths worldwide [1]. In Japan, the number of cases of cervical cancer increases from a woman's 20s and reaches its peak in her 40s [2]. About 16.8 females per 100,000 are diagnosed with cervical cancer each year, which means approximately 11,000 new cases are reported annually [2]. Cervical cancer is the second leading cause of cancer-related deaths among females aged 25 to 40 years, with approximately 2,900 deaths reported each year [2]. The human papillomavirus (HPV) vaccine is considered one of the most effective ways to prevent cervical cancer [1,2]. HPV vaccination began in April 2013, but Japan stopped its active recommendation in June 2013 [3]. In 2022, Japan resumed active HPV vaccine recommendations and also started a catch-up vaccination program [3]. However, HPV vaccination rates remain low and are a major concern [4-6]. There is also concern about the lack of vaccination among men [3,7,8].

There have been many reports on people's perceptions of the HPV vaccine [7-15], but most of these are based on selective surveys. On the other hand, text mining is a technique used in qualitative research and has been applied to investigate perceptions and ideas about the COVID-19 vaccine and other diseases [16-19]. Text mining automatically extracts words from text data and breaks them down into sentences or individual words. It then organizes this information in an easily understandable form. Using a variety of statistical methods, text mining can help identify patterns and relationships between words and sentences [16-19]. In our previous reports, we used text mining to analyze medical workers' perceptions of COVID-19 vaccines [16,17]. We also compared perceptions about COVID-19 and influenza using Twitter® data [18]. These studies show that text mining can provide new insights into specific words and their relationships that may be missed by traditional analysis methods.

Although text mining is useful, there are not many studies that use this method to analyze perceptions of the HPV vaccine [20-22]. Therefore, to promote future strategies for preventing cervical cancer, we investigated the perceptions of the HPV vaccine among students in a healthcare university in Okayama city, Japan.

## Materials and methods

Materials

**Figure 1:**
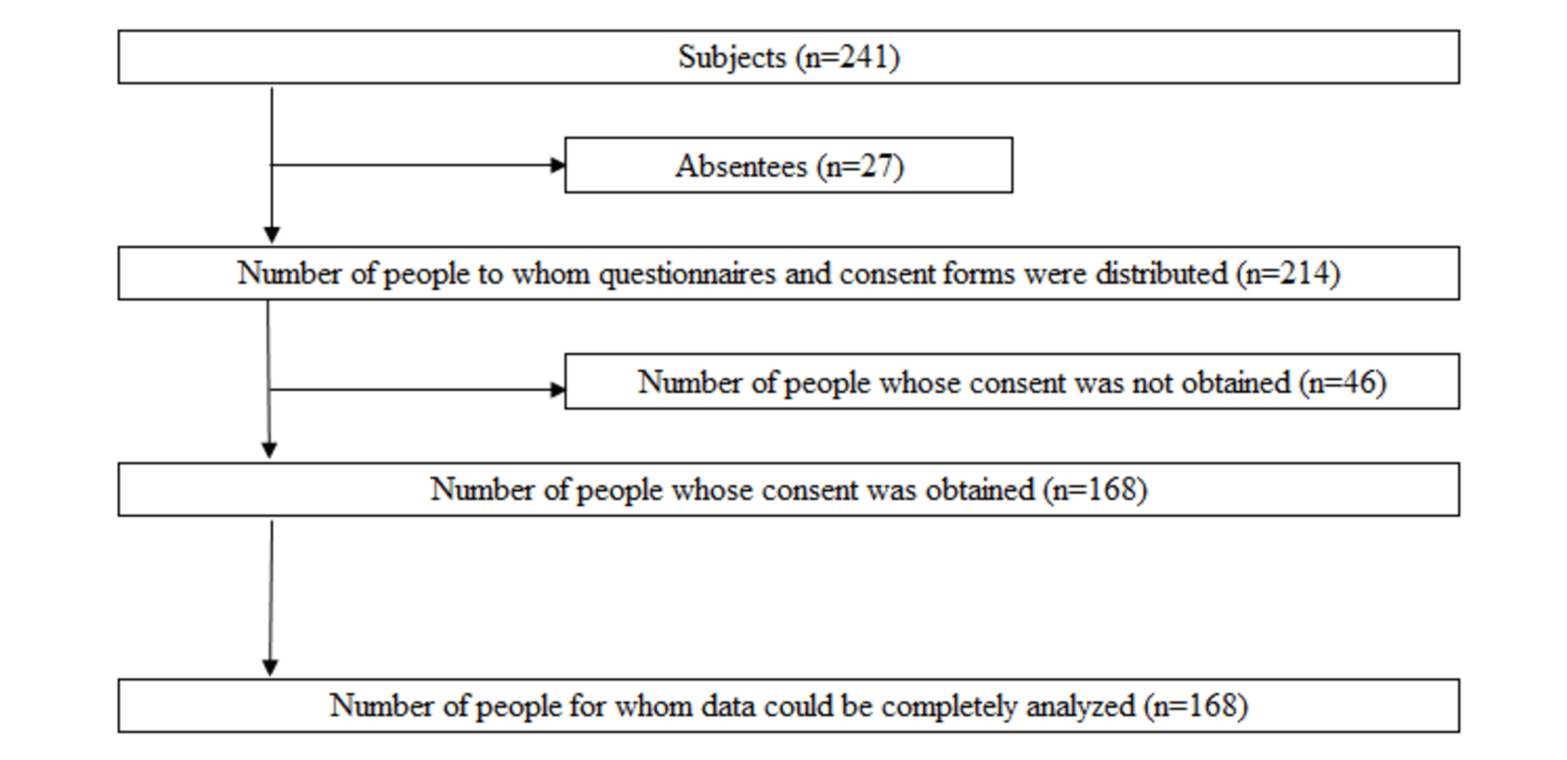
Selection process for the target population

A total of 168 (69.7%) of 241 students (94 male students and 74 female students, median age: 20 (minimum: 18, maximum: 27)) from a healthcare professional university in Okayama prefecture, Japan were included in this cross-sectional study. They were mainly physical and occupational therapy students. We did not calculate the sample size, and all students at Healthcare Professional University were primarily included. Exclusion criteria were those who were absent or could not give consent. After written informed consent was obtained, those who responded were enrolled (Figure 1).

Questionnaire

Between June 10, 2024, and July 29, 2024, we asked the respondents the following question: “What do you think about HPV vaccination? Please answer in a sentence.” by means of an open-ended questionnaire. The respondents were also asked about their sex, age, year, HPV vaccination status (yes or no), and cervical cancer screening status (yes or no). Furthermore, knowledge about HPV was assessed with the following questions: “Do you know the term HPV?” (yes or no), “Do you know that one of the causes of cervical cancer is HPV?” (yes or no), and “Do you know that the HPV vaccine is also effective for males?” (yes or no). These questionnaires were administered using QR codes and Google Forms® [23]. The students answered the questions using their own devices. The questionnaires took between 5 and 10 minutes to complete.

Statistical analysis

Data are presented in terms of the number of respondents and year (by male and female) and median age. For the question about HPV, results are presented as the number of subjects and percentages by total and sex. Fisher's exact test was used, and p < 0.05 was set as the level of statistical significance. Data were analyzed using the statistical software JMP Pro 17 (SAS Institute Inc., Cary, NC, USA).

Using the methodology of our previously reported studies [16-19], self-reported text passages on perceptions of HPV vaccination were analyzed using text-mining software (KH Coder 3.0, Koichi Higuchi, Japan) [24,25]. Words were automatically extracted from the sentences collected by Google Forms® using KH Coder to create a list of frequently appearing words. KH Coder was run with default settings to eliminate as much bias as possible due to analyst opinion. The morphological analysis dictionary also used default settings. The word “cervical cancer” was set as the coding rule for the analysis. Next, using correspondence analysis [16-19,24], which enables the visualization of relationships between words in scatter plots, we conducted analyses based on several factors: by HPV vaccination status, by HPV vaccination status and whether the respondent knew the term HPV, by HPV vaccination status and whether the respondent knew that HPV was a cause of cervical cancer, by HPV vaccination status and whether the respondent knew that the HPV vaccine is also effective for males, and by HPV vaccination status and cervical cancer screening status. Finally, the extracted words were translated into English using DeepL® translation (DeepL, Cologne) [26]. In addition, DeepL® translation and ChatGPT® (OpenAI Incorporated, Mission District, San Francisco, United States) were also used for some of the English editing.

## Results

Clinical profiles

The clinical profiles of the subjects of this study are listed in Table 1. There were 94 male students (56.0%) and 74 female students (44.0%), ranging in age from 18 to 27 years with a median of 20 years. 

**Table 1:** Clinical profiles of enrolled subjects (n=168)

		Male	%	Female	%	Total	%
Year	1st year	5	5.3	1	1.4	6	3.6
	2nd year	25	26.6	23	31.1	48	28.6
	3rd year	28	29.8	25	33.8	53	31.5
	4th year	36	38.3	25	33.8	61	36.3

Questions about HPV

Questions about HPV (Table 2) included: “Have you ever been vaccinated against human papillomavirus (HPV)?” and “Do you know the term human papillomavirus (HPV)?”. Female students responded “yes” to these questions significantly more frequently than males. Regarding the questions "Do you know that one of the causes of cervical cancer is human papillomavirus (HPV)?" and “Do you know that the human papillomavirus (HPV) vaccine is also effective for males?”, there were no significant differences between male students and female students for both questions. The question, “Have you ever been screened for cervical cancer?” (asked to female students only) received 12 responses for “yes” and 62 responses for “no.”

**Table 2:** Results of questions about HPV Fisher's direct probability test (*p*< 0.05) Values in bold indicate *p*< 0.05

Questionnaire	Total	%		Male	%	Female	%	p
Have you ever been vaccinated against human papillomavirus (HPV)?	13	7.7	Yes	1	1.1	12	16.2	0.0003
	155	92.3	No	93	98.9	62	83.8	
Do you know the term human papillomavirus (HPV)?	101	60.1	Yes	49	52.1	52	70.3	0.0183
	67	39.9	No	45	47.9	22	29.7	
Do you know that one of the causes of cervical cancer is human papillomavirus (HPV)?	80	47.6	Yes	41	43.6	39	52.7	0.3523
	88	52.4	No	53	56.4	35	47.3	
Do you know that the human papillomavirus (HPV) vaccine is also effective for males?	16	9.5	Yes	7	7.4	9	12.2	0.4280
	152	90.5	No	87	92.6	65	87.8	
Have you ever been screened for cervical cancer?	12	16.2	Yes	－	－	12	16.2	－
	62	83.8	No	－	－	62	83.8	

Frequently occurring words

Text mining analysis was performed. The total number of words obtained for female respondents was 1,232, with “think” (24 words) being the most common, followed by “vaccine” (23 words), “side effects” (23 words), “vaccination” (21 words), “frightening” (19 words), and “inoculate” (14 words) (Table 3).

**Table 3:** Frequently occurring words among female respondents (1,232 total words)

Rank	Word	Number of words	%	Rank	Word	Number of words	%
1	Think	24	0.95	11	Man	7	0.28
2	Vaccine	23	0.91	12	Little	6	0.24
2	Side effects	23	0.91	13	After effect	5	0.20
4	Vaccination	21	0.83	14	Rumor	4	0.16
5	Frightening	19	0.75	14	Consider	4	0.16
6	Inoculate	14	0.55	14	Myself	4	0.16
7	Take	13	0.51	15	Impression	3	0.12
7	Hear	13	0.51	15	Say	3	0.12
9	Be understood	10	0.39	15	Now	3	0.12
10	Cervical cancer	9	0.35	15	Few	3	0.12

The total number of words obtained for male respondents was 1,271, with “think” (39 words) being the most common, followed by “vaccine” (17 words), “vaccination” (15 words), “be understood” (12 words), “image” (10 words), and “inoculate” (10 words) (Table 4).

**Table 4:** Frequently occurring words among male respondents (1,271 total words)

Rank	Word	Number of words	%	Rank	Word	Number of words	%
1	Think	39	3.07	11	Consider	6	0.47
2	Vaccine	17	1.34	11	Take	6	0.47
3	Vaccination	15	1.18	11	For the first time	6	0.47
4	Be understood	12	0.94	11	Side effect	6	0.47
5	Image	10	0.79	11	Validity	6	0.47
5	Inoculate	10	0.79	11	Prevention	6	0.47
5	Know	10	0.79	17	Male	5	0.39
5	Necessary	10	0.79	17	Especially	5	0.39
5	Hear	10	0.79	17	Good	5	0.39
10	Cervical cancer	8	0.63	20	Man	4	0.31

Correspondence analysis

**Figure 2:**
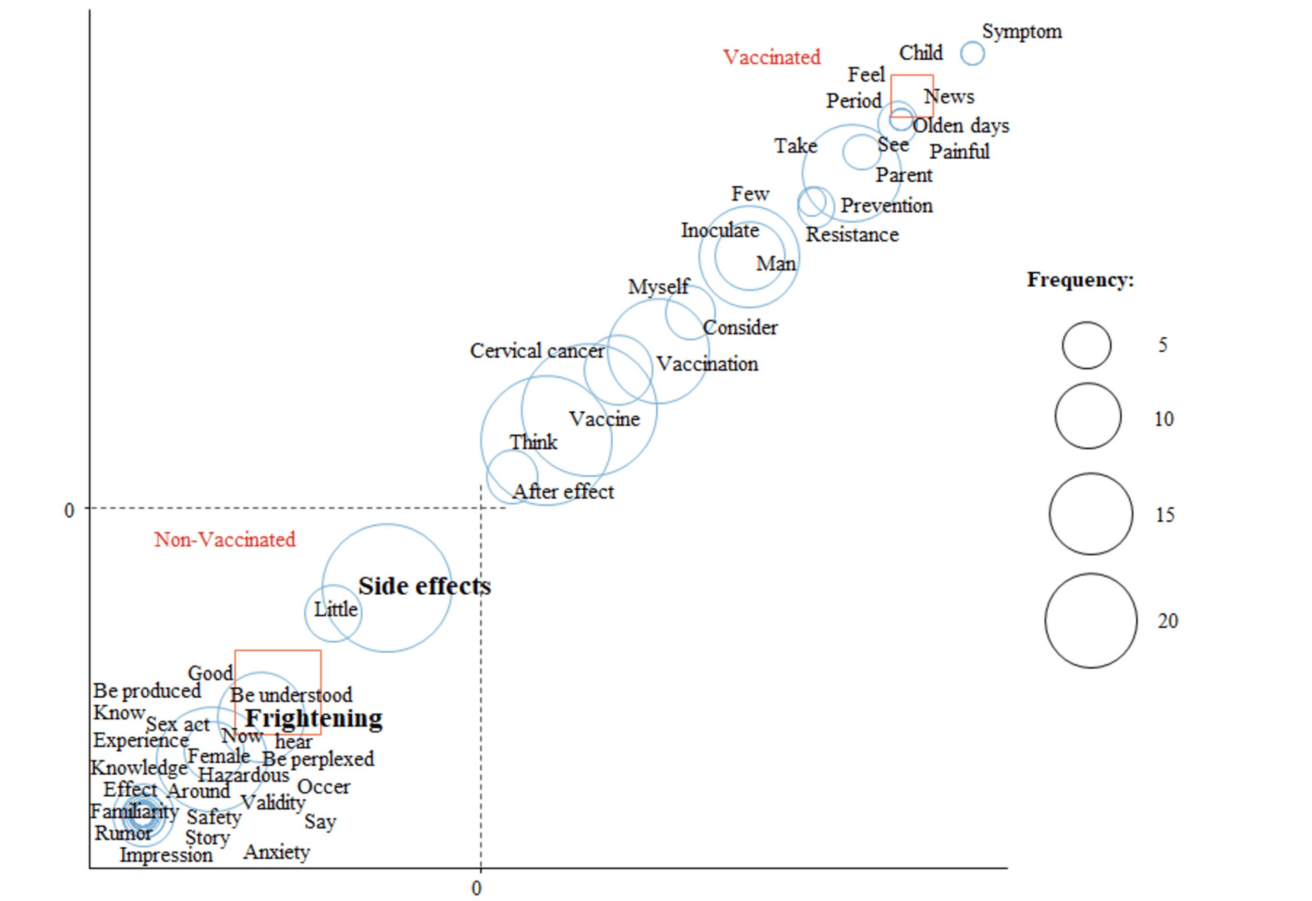
Correspondence analysis by HPV vaccination status in female students

A correspondence analysis was conducted for female students. Figure 2 shows the results of the correspondence analysis analyzed by HPV vaccination status, with “side effects” and “frightening” for the unvaccinated, and “vaccine” and “vaccination” for the vaccinated as characteristic words.

**Figure 3:**
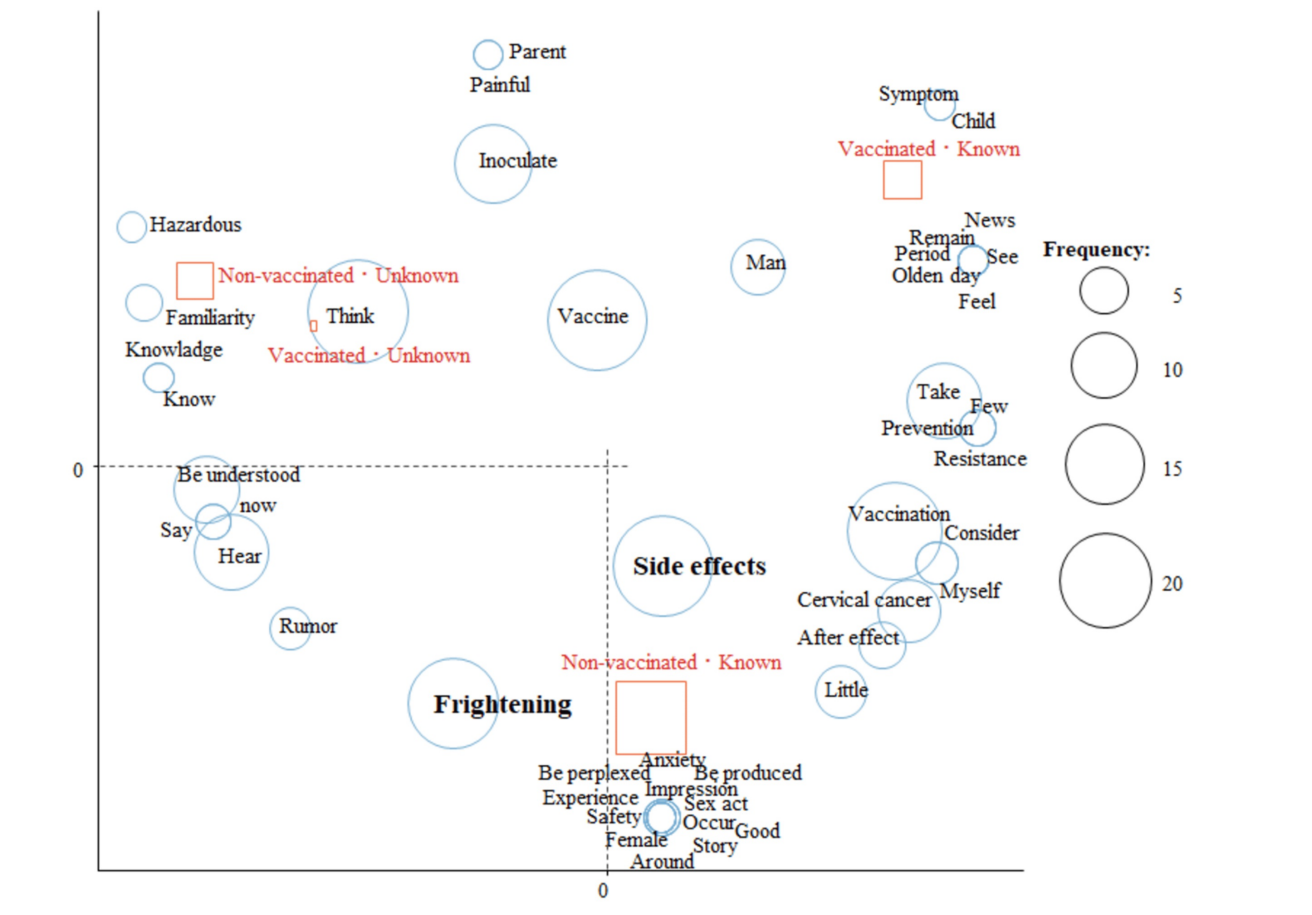
Correspondence analysis by HPV vaccination status and familiarity with the term HPV in female students

In the analysis of HPV vaccination status and whether they knew the word HPV, “side effects” and “frightening” were characteristic words for those who had not been vaccinated and knew the word HPV (Figure 3).

**Figure 4:**
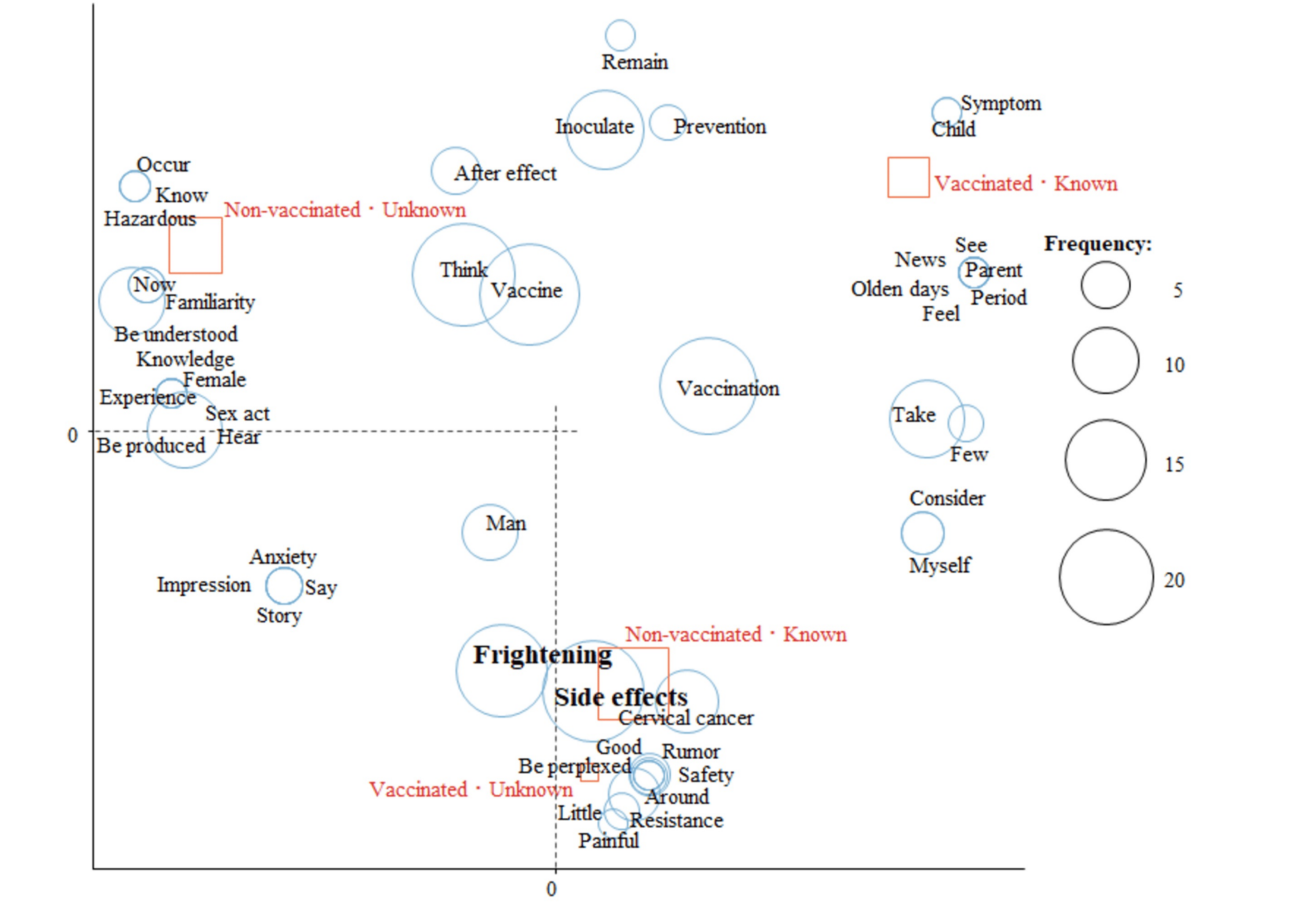
Correspondence analysis by HPV vaccination status and knowledge of HPV as a cause of cervical cancer in female students

In the analysis by HPV vaccination status and whether or not the respondents knew that HPV was a cause of cervical cancer, “side effects” and “frightening” were characteristic words for those who had not been vaccinated against HPV and knew that HPV was a cause of cervical cancer (Figure 4).

**Figure 5:**
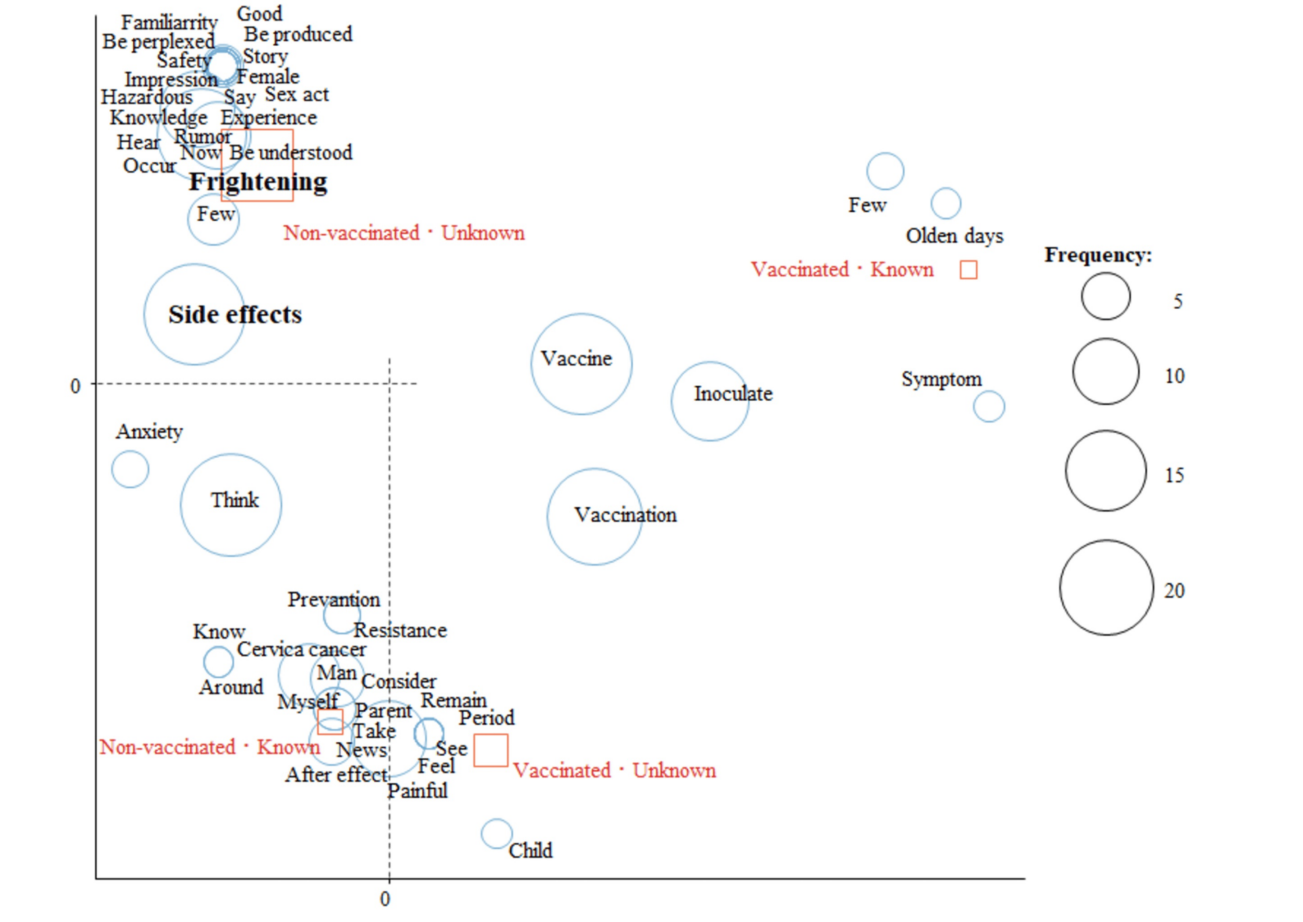
Correspondence analysis by HPV vaccination status and knowledge that HPV vaccine is also effective for men among female respondents

In the analysis of whether the respondents were vaccinated against HPV and whether they knew about the HPV vaccine being effective for men, those who were unvaccinated and did not know that the HPV vaccine is also effective for men were characterized by “side effects” and “frightening” (Figure 5).

**Figure 6:**
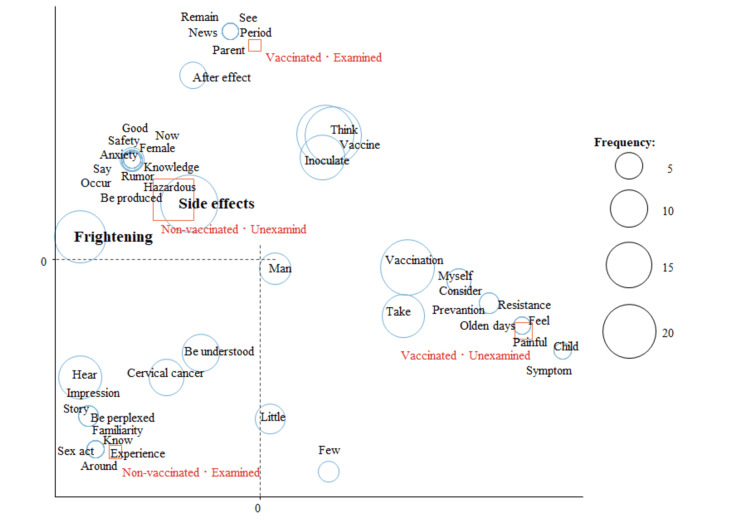
Correspondence analysis by HPV vaccination status and cervical cancer screening status

In the analysis of HPV vaccination status and cervical cancer screening status, “side effects” and “frightening” were characteristic words for those who had not been vaccinated and had not undergone screening (Figure 6).

## Discussion

In this study, we examined the perceptions of HPV vaccination among university students training to become healthcare professionals in Japan. HPV vaccination coverage among female students was comparatively low (16.2%). We found differences in the perceptions of HPV vaccination between vaccinated and unvaccinated women. Among the unvaccinated, “side effects” and “frightening” were especially characteristic among those who knew the term HPV, knew that HPV was a cause of cervical cancer, and had not yet undergone screening.

Currently, 132 countries have introduced the HPV vaccine [27]. HPV vaccination coverage in the G7 countries is over 80% in Canada; about 70% in the United Kingdom; and about 50% in Germany, Italy, the United States, and France [27]. On the other hand, Japan's HPV vaccination rate is about 35%, which is extremely low compared with other G7 countries [27]. Especially, the HPV vaccination rate among women born between 1997 and 2010 is very low (less than 10%) [28]. In Canada and the United Kingdom, routine HPV vaccination is offered to men as well as women [27]. Other than Canada and the United Kingdom, there are 75 countries that offer routine vaccination for both men and women; however, in Japan, routine HPV vaccination is still available only for men [27,28]. The HPV vaccination rate among female students in this survey was 16.2%. Students who are training to become healthcare professionals are likely to be a group that is more health-conscious than a typical population of young people; thus, we found HPV vaccination coverage rates that were higher than previous studies in Japan, but still lower than in other countries.

There are several previous studies that administered questionnaires to assess perceptions of HPV vaccination [9-13]. Aldawood et al. conducted a survey among Saudi university students to assess their awareness and knowledge of HPV vaccination and to investigate predictors associated with vaccine avoidance [9]. Radwan et al. surveyed Saudi women aged 18-49 years about their knowledge and awareness of cervical cancer and reported that knowledge and awareness were very low [10]. Faqeeh et al. surveyed the knowledge and awareness of HPV in Saudi Arabia and reported that despite positive attitudes, it had not led to HPV vaccination [11]. Wu et al. reported quantitatively on knowledge and perceptions of HPV among Chinese men [12]. In Japan, Nakagawa et al. conducted an internet-based questionnaire survey on the opinions of HPV unvaccinated women regarding catch-up vaccination [13]. These studies all used multiple-choice questions.

Some reports have used text mining to assess perceptions of HPV vaccination [20-22]. Okuhara et al. used text mining to analyze the content of Japanese websites on HPV vaccination and reported that the impression of HPV vaccination was influenced by information available on websites [20]. In another study, Okuhara et al. analyzed newspaper articles by text mining and reported that the words changed significantly before and after the HPV vaccination crisis (temporary suspension of active HPV vaccine recommendation) [21]. Luo et al. reported the impact of social media by using text mining to analyze opinions about HPV vaccination on Twitter® for a 10-year period from 2008 to 2017 [22]. These studies suggest that the use of text mining is more effective in analyzing perceptions and evaluating thoughts about HPV vaccination. In this study, we used text mining to evaluate the perceptions of HPV vaccination of students at a university for training healthcare professionals. Our findings showed that the words “side effects” and “frightening” were particularly characteristic among those who were unvaccinated for HPV, especially among those who knew the term HPV, knew that HPV was a cause of cervical cancer, and had not been screened. Our findings suggest that HPV vaccination could be further promoted by providing appropriate information on “side effects” and “frightening” aspects of HPV vaccination. Significantly more women (70.3%) than men (52.1%) knew about the term HPV and 43.6% of male students and 52.7% of female students knew that HPV was a cause of cervical cancer; however, only 7.4% of male students and 12.2% of female students knew that the HPV vaccine was also effective for men. Given these considerations, our findings suggest the necessity of providing basic information early. Although the HPV vaccine is currently not actively recommended for men in Japan, it may be effective in raising awareness of the term HPV itself from the view of preventing HPV infection among women. Zhou et al. [29] and Felsher et al. [30] reported that awareness-raising activities to increase correct knowledge for both men and women are important to improve HPV vaccination coverage. Moreover, Ishimoto et al. reported that awareness campaigns that consider the characteristics of men and women are important for promoting HPV vaccination [3].

This study has some limitations. First, this was a small cross-sectional study at a single facility in Okayama city, not a longitudinal study. Second, the subjects were students at a university for training healthcare professionals; thus, they are a population that is presumed to be relatively more health-conscious than the general student population. Third, the subjects who participated in this study were of the generation whose HPV vaccination recommendations were suspended from 2013 to 2022, which may have affected their perceptions and knowledge. Fourth, only 69.7% of the total number of subjects participated in this study, and there was a wide variation in age and year level. Thus, there are many remaining issues to be addressed in this survey, and the results of this study may not be applicable to Japan as a whole. Nevertheless, we are confident that the results obtained in this study will provide useful information for future HPV vaccination promotion efforts.

## Conclusions

When analyzed according to HPV vaccination status, the rates of HPV vaccination among healthcare professional university students in Okayama, Japan, were found to be comparably low. Our findings highlight the pressing need to provide accurate and comprehensive information regarding the “side effects” and potentially “frightening” aspects associated with HPV vaccination, particularly targeting those who remain unvaccinated. This includes especially individuals who are already familiar with the term HPV, understand its connection to cervical cancer, and have not yet undergone screening for cervical cancer. By addressing these concerns, we believe that our results will contribute valuable insights for developing more effective HPV vaccination strategies in the future.

## Appendices

**Table 5:** Questionnaire on impressions of HPV vaccine

Questionnaire				
What do you think about human papillomavirus (HPV) vaccination? Please answer in a sentence.				
Have you ever been vaccinated against human papillomavirus (HPV)?	Yes	No		
Do you know the term human papillomavirus (HPV)?	Yes	No		
Do you know that one of the causes of cervical cancer is human papillomavirus (HPV) ?	Yes	No		
Do you know that the human papillomavirus (HPV) vaccine is also effective for males?	Yes	No		
Have you ever been screened for cervical cancer?	Yes	No		
Sex	Male	Female		
Age (years)				
Year	1st year	2nd year	3rd year	4th year
